# Effect of saccade automaticity on perisaccadic space compression

**DOI:** 10.3389/fnsys.2015.00127

**Published:** 2015-09-10

**Authors:** Michele Fornaciai, Paola Binda

**Affiliations:** ^1^Department of Neuroscience, Psychology, Pharmacology and Child Health, University of FlorenceFlorence, Italy; ^2^Department of Translational Research on New Technologies in Medicine and Surgery, University of PisaPisa, Italy

**Keywords:** eye movements, saccades, perisaccadic mislocalization, spatial compression, practice

## Abstract

Briefly presented stimuli occurring just before or during a saccadic eye movement are mislocalized, leading to a compression of visual space toward the target of the saccade. In most cases this has been measured in subjects over-trained to perform a stereotyped and unnatural task where saccades are repeatedly driven to the same location, marked by a highly salient abrupt onset. Here, we asked to what extent the pattern of perisaccadic mislocalization depends on this specific context. We addressed this question by studying perisaccadic localization in a set of participants with no prior experience in eye-movement research, measuring localization performance as they practiced the saccade task. Localization was marginally affected by practice over the course of the experiment and it was indistinguishable from the performance of expert observers. The mislocalization also remained similar when the expert observers were tested in a condition leading to less stereotypical saccadic behavior—with no abrupt onset marking the saccade target location. These results indicate that perisaccadic compression is a robust behavior, insensitive to the specific paradigm used to drive saccades and to the level of practice with the saccade task.

## Introduction

Saccades pose a major perceptual problem in that each new eye movement changes the mapping of external objects on the retina. While the visual system is usually able to overcome this challenge and ensure stable and seamless perception, there are conditions where vision is disrupted during saccades: for example, when visual stimuli are brief, flashed in and out of view within some 100 ms before or after the onset of the saccade.

These briefly presented stimuli are subject to systematic perceptual distortions, which include a transient underestimation of magnitude (or compression) that affects space, time and numerical quantities (reviewed in Burr et al., 2010). Specifically, spatial localization is biased for stimuli that are briefly presented around the saccade onset, leading to a compression of visual space toward the target of the saccade (Ross et al., 1997; Lappe et al., 2000) or, under special circumstances (e.g., complete darkness), producing a uniform shift of perceived positions in the saccade direction (Honda, 1989). Time as well as space is distorted; stimuli flashed at saccade onset are perceived as occurring at a systematically later time point (Binda et al., 2009) and the temporal distance of pairs of flashed perisaccadic stimuli is underestimated or “compressed” (Morrone et al., 2005). Compression, i.e., underestimation of the distance between stimuli along some perceptual dimension, extends even to abstract constructs such as numerical magnitude, affecting both analogic and symbolic representations of numbers that are briefly presented at the time of saccades (Binda et al., 2011, 2012).

It is important to note that all these distortions are never observed in everyday life, for two main reasons. First, illusions only occur for briefly flashed stimuli, which are very rare in natural scenes. Second, and most importantly, illusions vary widely with the time of flash occurrence relative to the onset of a saccade. As a consequence, perisaccadic distortions are typically observed in laboratory conditions that encourage production of highly stereotyped eye movements: saccades directed to one or few target locations, repeated thousands of times so as to focus data collection in the short perisaccadic temporal window where illusory perceptions occur. These conditions might differ in important ways from natural eye movement behavior, especially considering that different types of saccades are associated with different patterns of brain activity (Johnston and Everling, 2008; McDowell et al., 2008).

In particular, saccade control recruits three areas, the Lateral Intra-Parietal (LIP) cortex, the Frontal Eye Fields (FEF) and the Superior Colliculus (SC), which are also involved in the representation of perisaccadic visual space—as indicated by the fact that visual receptive fields change transiently at about the time of a saccade in LIP (Duhamel et al., 1992), FEF (Umeno and Goldberg, 1997; Sommer and Wurtz, 2006; Zirnsak et al., 2014) and SC (Walker et al., 1995). Modulation of activity in all three areas has been observed in experiments comparing the execution of saccades with different levels of volitional control or automaticity. Fronto-parietal activity associated with saccade execution differs depending on whether saccades are specified based on symbolic instructions (e.g., anti-saccades) or spontaneously triggered by the presentation of peripheral targets (targeting saccades)—as observed in several neuroimaging studies of human subjects (McDowell et al., 2008) and specifically in the FEF (Everling and Munoz, 2000) and LIP (Gottlieb and Goldberg, 1999) of non-human primates. Both FEF (Bruce and Goldberg, 1985) and LIP (Gottlieb et al., 1998) activity depends on the salience of the saccade target stimulus, with enhanced responses to salient visual transients (e.g., abrupt onset of a stimulus) targeted by spontaneous saccades. Although they are a simple and spontaneous behavior, saccades targeting visual transients can be automatized with practice. Repetition of saccades in one direction over hundreds of trials leads to shorter and less variable saccade latencies (Basso and Wurtz, 1998; i.e., saccade reaction times), and this is accompanied by stronger neural activity preceding saccade execution in SC (Basso and Wurtz, 1997, 1998). These changes occur in the intermediate layers of the SC, which project to the FEF and have been shown to carry a “corollary discharge” of the eye movement command—that is, a copy of the eye movement command that may help maintaining a stable representation of space in spite of the displacement of retinal images caused by the saccade (Sommer and Wurtz, 2002, 2006, 2008). Thus, there is evidence that the level of automaticity with which saccades are executed has an impact on neural activity in just those areas that play a key role in the representation of visual space. This raises the question whether visual space distortions observed during saccades are specifically associated with the execution of highly stereotyped saccades—rather than spontaneous, natural phenomena.

Here, we set out to directly address this question, using two experimental approaches. In the first, we tested the effect of practice on a repetitive saccade task. We compared a group of expert participant with a history of several thousands trials on the specific saccade task we used, with a group of novices, who had never participated in eye-movement related research, and practiced the saccade task over the course of the experiment. Second, we studied how the experts’ localization performance changed when the saccade task was modified to increase the level of volitional control. We changed the stimulus display so that the saccade target location was marked by a steadily visible stimulus, and subjects initiated saccades upon hearing a sound. The removal of the peripheral onset and the added complexity of the task, which no longer exploited our spontaneous tendency to saccade at a sudden peripheral onset, should lead to modification of saccade parameters—notably, latency (Walker et al., 2000; Rolfs and Vitu, 2007).

Our prediction is that, if perisaccadic compression is specifically associated with the repetitive execution of stereotypical saccades, it should be reduced in conditions that promote the variability of saccade parameters, and in novice observers—with an idiosyncratic pattern of perisaccadic mislocalization that gradually normalizes with practice.

## Materials and Methods

### Subjects

Twenty-four subjects participated in the experiment, after giving their written informed consent. Sixteen of the subjects were completely inexperienced in psychophysical experiments involving eye movements (“novices”) and were tested with the basic “abrupt onset” paradigm; data collection started immediately after the instructions were given (no practice trial). The other eight were “expert” observers (with a history of >1000 trials in experiments involving eye movements and including the two authors); these were tested with both the “abrupt onset” and a less usual “steady-on” paradigm (described below). All subjects reported normal or corrected to normal vision. Experimental procedures were approved by the local ethics committee [Comitato Etico Pediatrico Regionale—Azienda Ospedaliero-Universitaria Meyer—Firenze (FI)] and are in line with the declaration of Helsinki.

### Apparatus

The experiment was performed in a quiet and dark room. Subjects sat in front of a monitor screen (40 × 30 cm) at a distance of 57 cm, with their head stabilized by a chin rest. Stimuli were generated using the PsychoPhysics Toolbox routines (Brainard, 1997; Pelli, 1997) for Matlab (MatLab r2010a, The Mathworks, inc.) and presented on a CRT monitor (Barco Calibrator Line) with a resolution of 800 × 600 pixel and a refresh rate of 120 Hz, driven by a Mac Pro 4.1.

Eye movements were monitored in-synch with visual presentations using the EyeLink 1000 system (SR Research, Canada) and the Eyelink toolbox for Matlab (Cornelissen et al., 2002). Eye position and pupil diameter of the left eye were measured with a frequency of 1000 Hz by means of an infrared sensor mounted below the screen, which allowed for unrestrained binocular viewing of the display. At the beginning of the experimental session, a standard 13-point calibration routine was performed.

### Stimuli

Stimuli were presented against a homogeneous red background (Commission International d’Eclariage coordinates: *x* = 0.624, *y* = 0.343; luminance = 23 cd/m^2^); the localization probe was a green vertical bar (CIE coordinates: *x* = 0.292, *y* = 0.597; luminance = 60 cd/m^2^), 1 degree wide, straddling the full screen height (30 degrees). It was presented for a single monitor frame, at variable horizontal locations and timings.

### Task and Procedure

**Figure 1:**
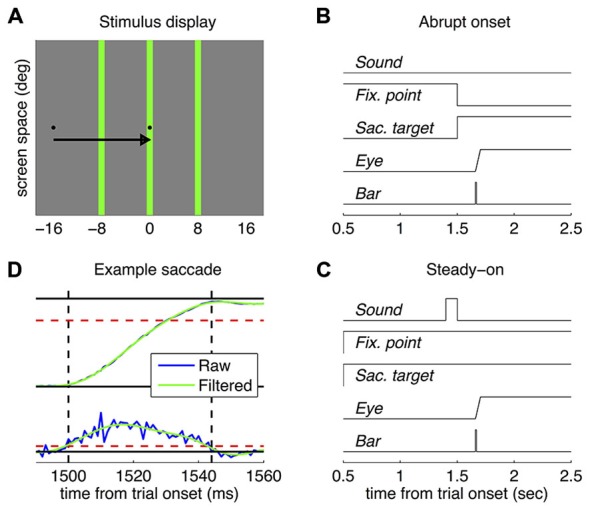
**Methods. (A)** Approximately in-scale representation of the stimulus display; the arrow shows the required saccade (not part of the display) and the three vertical green bars indicate the tested positions (only one was tested in each trial). **(B,C)** Timecourse of presentations in the abrupt onset target and the steady-on target paradigm respectively. **(D)** Example eye position (top) and velocity traces (bottom), before and after the Fourier filtering. Dashed red lines show the velocity threshold for detection of saccade onset and offset (marked by the vertical dashed black lines) and the exclusion criterion for saccade amplitude (the saccade had to be >12 degrees, 3/4 of the required saccade amplitude).

In all cases, subjects made rightward saccades from a small black square located 16 degrees left of screen center (the fixation point) to an identical black square presented at the screen center (the saccadic target)—see Figure 1A. The timecourse of presentation of the two stimuli, however, differed in the two tested paradigms—see Figures 1B,C. In the “abrupt onset” paradigm, the saccade target appeared after a variable delay (1500 ± 100 ms) and simultaneously with the extinction of the fixation point, giving the impression of a point that jumped between the two locations and that the subjects were instructed to promptly follow with their eyes. In the “steady-on” paradigm, both the fixation point and the saccade target remained always visible; subjects were signaled to start a saccade by a sound (100 ms white noise burst, approximately 80 dB). In all cases, the probe bar was presented at one of three horizontal positions, randomly intermixed across trials: at −8, 0 or +8 degrees relative to the saccadic target (negative meaning leftward; Figure 1A). About 1 s after the probe bar presentation, the mouse cursor appeared at a random position (drawn from a circular Gaussian distribution with mean at screen center and standard deviation of 4 degrees) and subjects adjusted it to match the perceived position of the bar. In the rare cases where subjects failed to detect the bar (2.4 ± 0.7% of trials), they were instructed to click in the bottom left corner of the screen—so that the trial could be discarded from the analyses. Response collection triggered the start of the next trial. Trials were administered in blocks of 24 separated by short breaks; each subject was tested in at least ten blocks per experiment. In the first five blocks, the probe bar was presented immediately upon detection of the saccade onset—calculated online as the first of two consecutive time points where horizontal eye velocity exceeded 100 deg/s. In the rest of the blocks, the time of bar was defined *a priori* based on the subject’s saccade latency (median across all previous trials in the experiment) and an average intended delay of ±50 ms. Triggering in the first half of the trials was aimed to avoiding that the early trials of novice subjects be wasted over non-perisaccadic bar presentations, maximizing the probability to reveal practice effects. However, for simplicity, the same procedure was also adopted with expert subjects. All blocks also included a minority of trials where the signal for starting the saccade was withheld and localization was measured during fixation.

### Data Analyses

An offline analysis examined the horizontal eye position traces. Visual inspection indicated some contamination from high-frequency noise. We therefore proceeded to filter the traces in the frequency domain (applying Fast Fourier Transform (FFT) to each trace, multiplying the frequency spectrum by a Gaussian centered at 50 Hz with standard deviation of 10 Hz respectively, then reconverting the traces to the time domain via the inverse FFT). Figure 1D shows the raw and filtered traces for eye position and velocity for a representative trial. Filtering had virtually no impact on the estimation of any saccade parameter except for peak velocity—the absolute values of which were of course higher for the unfiltered traces and less orderly related to saccade amplitude. We then re-estimated saccade onset (first of two samples exceeding 100 deg/s velocity) and consequently re-determined the time of probe-bar presentation relative to the saccade; we also estimated the other saccade parameters: offset (first of two samples falling below the 100 deg/s threshold), duration, amplitude and peak velocity. Trials were discarded from further analyses if no saccade could be detected (<1% in all cases), the saccade was anticipatory (negative latency, 8.6 ± 3% and 4.0 ± 2% in the “abrupt onset target” paradigm, for novices and experts respectively; 5.3 ± 2% for Experiments in the “steady-on target” paradigm) or smaller than 12 degrees (3/4 of the required amplitude, 7.2 ± 2%, 7.5 ± 4%, 5.4 ± 2%), leading to the exclusion of 18.4 ± 4%, 12.3 ± 6% and 11.0 ± 4% of trials.

**Figure 2:**
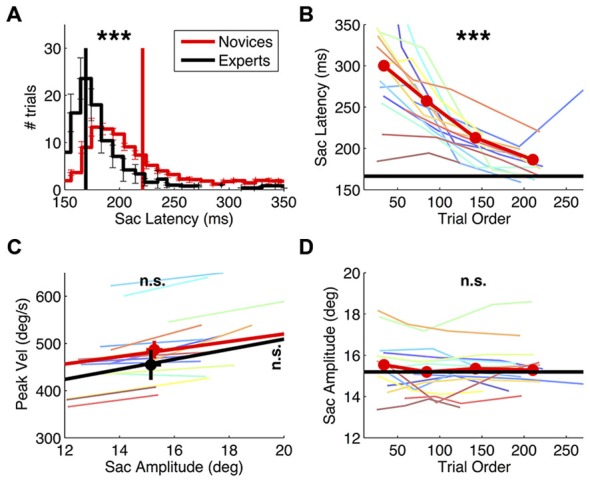
**Effect of practice on saccade behavior.** Saccade latency and main sequence in novice vs. expert observers and evolution of novices’ saccade behavior over the course of the experiment. **(A)** Saccade latency histogram, computed for the individual subjects and then averaged, with errorbars showing s.e. in each bin. Vertical lines mark the grand averages of saccade latency. Asterisks at the top of the panel report the result of a two-sample *t*-tests comparing the averages in the two groups (***p < 0.001). **(C)** Saccade main sequence, plotting peak velocity against amplitude. Colored lines give the best fit line for the individual novice subjects; thick red and black lines give the average fit for the two subjects groups, and symbols with errorbars show grand averages and their s.e.. The results of two-sample *t*-tests comparing the average peak velocity and amplitude in the two groups are marked on the right and at the top of the graph respectively (n.s. = not significant). **(B–D)** Saccade latency and amplitude for novice subjects, computed after ranking trials according to their presentation order and averaging values in the four quartiles; colored lines show the individual novice subjects, the thick red line gives the averages in the novices group; asterisks report the results of paired *t*-tests comparing the first vs. the last quartile. For reference, the average in the experts’ group in also shown (black line).

Localization performance in the valid trials was analyzed by sorting trials based on the time of bar presentation relative to saccade onset; for each tested position of the bar, average localization was computed in partially overlapping bins of 30 ms width (overlap: 20 ms); the curves averaged across observers are shown in Figure 2. We summarized localization performance by computing, in the same bins, a compression index and a shift index. These were estimated in Lappe et al. (2000): the compression index is the standard deviation of reported bar positions, normalized by the standard deviation of the actual bar positions; the shift index is the mean of the reported bar positions relative to the mean of the actual bar positions. Based on these definitions, a compression index of zero indicates maximal compression and one indicates no compression; a positive shift index indicates an overall bias in the direction of the saccade. We verified that using an alternative definition of compression and shift indices, e.g., slope and intercept of the reported vs. actual positions as in Zimmermann et al. (2014b), did not alter the results. Statistical comparisons across groups/conditions were performed on a subset of trials, where the bar was presented within 15 ms from the saccade onset (i.e., between −15 ms and +15 ms from saccade onset) leading to the maximum expected mislocalization. For the analysis of practice effects in novices, trials in this bin were sorted by presentation order and mislocalization indices and saccade parameters were computed in the first through fourth quartiles.

Besides performing ordinary statistical tests (*t*-tests) we evaluated their statistical power using Bayesian methods. Specifically, we computed the Bayes Factor (BF) following Dienes (2014). This requires entering a description of the data (*t* statistics and its standard error) and a description of the theory (the distribution of the statistics under the “alternative hypothesis”). We corrected the standard error of the *t*-statistics by 1 + 20/(df × df) as recommended for degrees of freedom df < 30. We only computed the BF for comparisons of compression index values, were the distribution of the statistics is easily defined: a uniform distribution bound within −1 and 1 (the difference between compression indices, which vary between 0 and 1). Conventionally, a BF larger than three implies support for the alternative hypothesis, BFs between 1/3 and 3 indicate weak or no evidence for either hypothesis and a BF smaller than 1/3 implies strong evidence in support of the null hypothesis.

## Results

Our first experiment compared perisaccadic localization in two groups of subjects: expert subjects (with long prior experience in experiments employing the same eye movement task, here referred to as the abrupt-onset paradigm) and novices (with no prior exposure to psychophysical tasks involving an eye movement).

We analyzed their eye movement performance—in terms of the main saccade parameters: peak velocity, amplitude and latency—and how these changed over the course of the experiment, i.e., with practice.

As expected, practice had a strong effect on saccade latencies. Their distribution differs markedly between groups (Figure 2A), being much longer in the novices (two-sample *t*-test: *t*_(22)_: 6.21, *p* < 0.001) and more variable from trial to trial (two-sample *t*-test on the standard deviation of saccade latency values: *t*_(22)_: 6.08, *p* < 0.001). Over trials, novices’ saccade latencies decreased (Figure 2B; paired *t*-test comparing latencies in the first vs. the fourth quartile of trials: *t*_(15)_: −6.05, *p* < 0.001) and so did their trial-by-trial variability (paired *t*-test: *t*_(15)_: −4.77, *p* < 0.001, not shown). At the beginning of the experiment, novices’ saccade latencies were almost twice as long as the experts’; within the approximately 200 collected trials, they normalized to values similar to the experts’ (though still slightly longer, two-sample *t*-test comparing saccadic latencies in experts vs. the novices’ last quartile of trials: *t*_(22)_: 2.41, *p* < 0.05).

Practice did not affect the other saccade parameters (Figures 2C,D). Saccade amplitude and peak velocity are comparable between novices and experts (two-sample *t*-test on amplitude values: *t*_(22)_: 0.27, *p* = 0.791; two-sample *t*-test on peak velocity values: *t*_(22)_: 0.96, *p* = 0.348) and so is the variability of these indices across trials (all *p* > 0.2). Moreover, neither parameter changed systematically over the course of the experiment (paired *t*-test comparing saccade amplitude in novices’ early vs. late trials: *t*_(15)_: −1.86, *p* = 0.083; peak velocity: *t*_(15)_: −0.37, *p* = 0.718).

Having established that practice had a robust effect on key descriptors of saccade behavior, namely saccade latency and its trial-to-trial variability, we went on to analyze its effect on localization performance.

**Figure 3:**
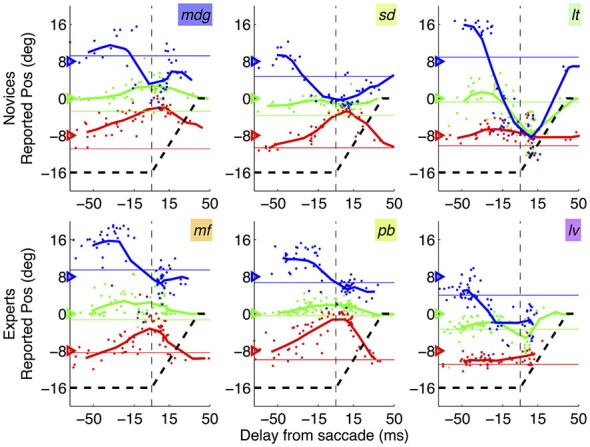
**Mislocalization curves in novices vs. experts.** Pattern of mislocalization in three representative novice subjects (top row) and three expert subjects (bottom row), all tested with the abrupt onset paradigm. Thick lines give running averages of localization judgments (averages in bins of 30 ms stepping by 10 ms) and dots show individual trials data. Subjects’ initials are given by the text insets, with colors corresponding to those used in Figures 2, 4 (novices) and 5 (experts). The triangles on the left *y*-axis indicate the veridical position of the bars; the black dashed line shows the trajectory of the saccade, from the fixation point (−16 degrees) to the saccade target (0 degree, or screen center).

Figure 3 shows results from three representative novice subjects and three experts (top and bottom rows respectively), plotting the average reported position of each tested position as function of the delay of bar presentation relative to the saccade onset. In all cases, the pattern of localization errors is consistent with the established phenomenon of perisaccadic compression—stimuli presented at about the saccade onset tend to be seen as compressed within a small spatial region. There is substantial inter-subject variability as to the “focus of compression”: bars may be seen as compressed toward the saccade target or left/right of it. However this variability, which was noted previously (Morrone et al., 1997), appears to be present in both novice subjects and experts—and it did not correlate with any of the analyzed saccade parameters, including saccade lading (see correlation analyses below).

In order to test for systematic differences of localization behavior between the two groups, we defined two indices of perisaccadic mislocalization: compression and shift index—as in Lappe et al. (2000).

**Figure 4:**
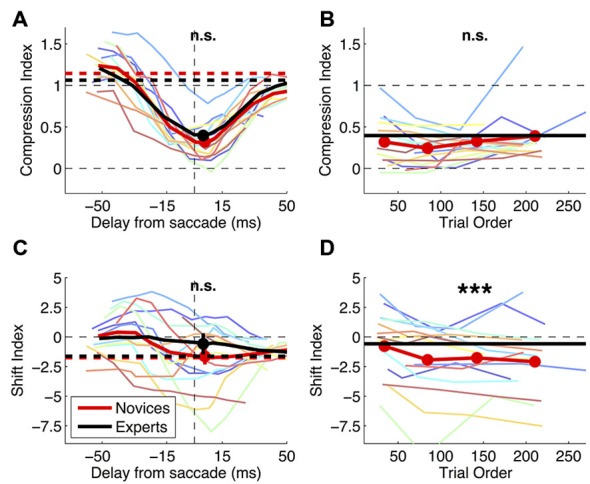
**Effect of practice on mislocalization indices.** Mislocalization in novice and expert subjects. **(A,C)** Compression and shift index plotted against the time of bar presentation. Thin colored lines show timecourses for the individual subjects (running averages as for Figure 3). Thick lines give the averages across subjects over the timecourse (continuous curves) and in fixation (dashed horizontal lines); filled symbols and their errorbars show averages and s.e. in the 30 ms bin spanning the saccade onset, which the *t*-tests compared (results given at the top of the graph; n.s. = not significant). **(B,D)** Evolution of compression and shift index values over the course of the experiment. Trials with the probe presented in the 30 ms straddling saccade onset were ranked according to their presentation order, and averages were computed in the four quartiles. Thin lines give the results for the individual subjects and red thick lines the average; for reference, grand-averages for the experts groups are also shown (black horizontal thick lines). Paired *t*-tests compared the first and last quartiles and results are marked at the top of the panels (n.s. = not significant, ***p < 0.001).

Figure 4A shows that novices and experts have closely matched values of compression index (two-sample *t*-test in the 30 ms straddling the saccade onset: *t*_(22)_: −0.93, *p* = 0.364). Bayesian statistics confirm that the lack of statistical significance is not due to lack of power: the BF given the observed data (*t*: −0.93, se: 0.09) is 0.16–a BF < 1/3 is conventionally interpreted as strong evidence for the null hypothesis, implying that compression is the same in novices and experts. Figure 4C shows shift index values, which are very variable across subjects (reflecting the idiosyncratic “focus of compression” seen in Figure 3). There is a tendency for more negative values in the novices group, but the difference does not reach statistical significance (two-sample *t*-test in the 30 ms straddling the saccade onset: *t*_(22)_: −0.96, *p* = 0.347).

As a more direct test for practice effects, Figures 4B,D show the variation of novices’ compression and shift indices over the course of the experiment. Comparing early vs. late trials (the first vs. the fourth quartile), we find no trend for the compression index to change with practice (paired *t*-test: *t*_(15)_: 1.08, *p* = 0.299). Again we use Bayesian statistics for the comparison of compression index values and find that the BF given the observed data (*t*: 1.08, se: 0.07) is 0.15, i.e., BF < 1/3 or strong evidence in support of the null hypothesis of no variation of compression index values. The shift index computed in the last quartile of the experimental trials is reliably more negative than at the beginning of the experiment (paired *t*-test: *t*_(15)_: −4.44, *p* < 0.001). However, this can hardly be interpreted as an effect of practice, given that the shift index in the novices grew progressively apart from the experts group.

In our second experiment, we focused on our expert observers and manipulated the saccade task. Rather than letting saccades be driven by the sudden appearance of the peripheral target, we had both the fixation and saccade target always visible (“steady-on”) and an auditory cue instructing the initiation of a saccade.

**Figure 5:**
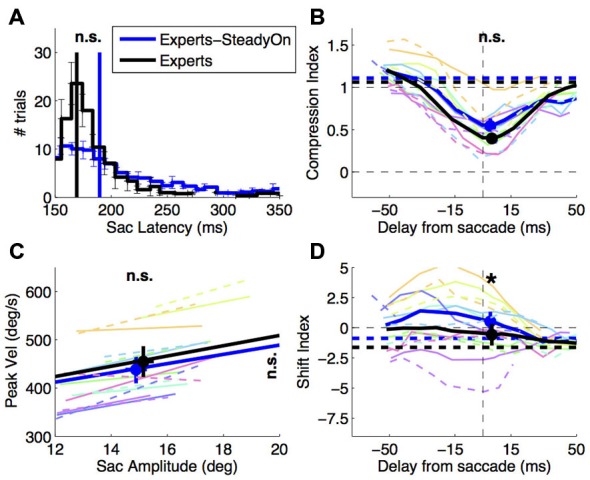
**Effect of saccade target presentation mode.** Saccade behavior and perisaccadic mislocalization in expert subjects, tested with the abrupt onset vs. the steady-on saccade target paradigm. **(A,C)** Saccade latency and saccade main sequence (same conventions as in Figures 2A,C); **(B,D)** compression and shift indices of perisaccadic mislocalization (same conventions as in Figures 4A,C). Paired *t*-tests compared the averages in the two conditions and the results (n.s. = not significant, *p < 0.05) are marked at the top of the panels **(A,B,D)** and at top and right of panel **(C)** (for values on the *x* and *y*-axis respectively). Thin colored lines in panels **(B–D)** show individual subject data: dashed for the abrupt onset, continuous for the steady-on saccade target paradigm.

Consistent with a more volitional nature of the saccade behavior in this condition, we find systematic differences in the saccade latency distribution (Figure 5B). There is an increase of trial-by-trial variability of saccade latency (paired *t*-test on the standard deviation of saccade latency values: *t*_(7)_: 5.22, *p* < 0.01). Contrary to Experiment 1, this is not accompanied by a significant change of average saccade latency (paired *t*-test: *t*_(7)_: 1.56, *p* = 0.163). All other saccade parameters are well matched across conditions. There is no difference of average saccadic amplitude (*x*-axis of Figure 5D, paired *t*-test on average saccade amplitude: *t*_(7)_: −1.45, *p* = 0.191) or its variability (paired *t*-test on the standard deviation of saccade amplitude values: *t*_(7)_: 1.12, *p* = 0.301) and no difference of average peak velocity (*y*-axis of Figure 5D; paired *t*-test: *t*_(7)_: −2.35, *p* = 0.051).

The change of saccade latency distribution was not accompanied by changes of perisaccadic compression—the compression index values does not differ significantly between conditions (Figure 5A, paired *t*-test in the 30 ms straddling the saccade onset: *t*_(7)_: 2.22, *p* = 0.062). However, we observe a tendency for shift index values to be more positive in the steady-on than in the abrupt onset target paradigm (paired *t*-test: *t*_(7)_: 3.23, *p* < 0.05)—the opposite of the trend observed comparing novices to experts.

In our last set of analyses, we pooled data from experts and novices collected with the abrupt-onset paradigm and analyzed the correlations between mislocalization indices and saccade parameters across subjects. Compression and shift indices do not correlate with any of the parameters we analyzed. In particular, there is no significant correlation between peak velocity and compression index values (Pearson’s linear correlation coefficient *R*_(22)_: 0.071, *p*: 0.741) and no correlation between the shift index (which describes the location of the “focus of compression” discussed in relation to Figure 2) and saccade landing position (*R*_(22)_: 0.050, *p*: 0.818).

## Discussion

Our two experiments compared the localization of briefly presented perisaccadic stimuli during different types of saccades, associated with different distributions of saccade latencies: spontaneous “targeting” saccades to a sudden peripheral onset, made with different levels of practice, or saccades instructed by an auditory stimulus. Our main finding is that the index describing perisaccadic compression (Lappe et al., 2000) remains similar in spite of large variations of saccade latency and its variability, suggesting that the level of automaticity with which the saccade task is performed does not influence the perisaccadic distortion of perceived space.

In the first experiment, we examined how perisaccadic compression indices vary as a function of practice on a simple targeting saccade task, in which saccades were repeatedly made to an abrupt peripheral onset occurring at the same location for the entire experiment. Novice observers (with no prior experience in saccade experiments) displayed longer and more variable saccade latencies compared with a pool of expert observers, and their behavior gradually converged towards the experts’ over the course of the experiment. Similar effect of practice on saccade latency has been reported for targeting saccades in non-human primates (Basso and Wurtz, 1998). We find that these large variations of saccade performance, both across subject groups and within novice subjects as function of practice, were not associated to any detectable difference of perisaccadic compression.

In the second experiment, we compared experts’ performance between the targeting saccade task described above and an atypical saccade task, where the saccade target was continuously visible and an auditory stimulus instructed subjects to initiate the saccade. Removing the sudden onset of a visual stimulus reduces its salience, affecting both behavioral responses (Yantis and Jonides, 1984) and neural responses in several areas—particularly areas tightly connected with eye movement control, such as LIP and FEF (Bruce and Goldberg, 1985; Gottlieb et al., 1998). In this task, we find that saccade have more variable latencies; this is consistent with stronger volitional control (associated with more variable behavior, Carpenter, 1999). However, contrary to what is typically found for voluntary saccades, the average saccade latency does not increase. This may be due to the relative simplicity of the task, which does not impose the time requirements of processing complex instructions and/or the meaning of symbolic cues that are often used for guiding voluntary saccades (Walker et al., 2000). Whichever the origin of the increased variability of saccade behavior, we found that this was not accompanied by a change of perisaccadic compression—which was indistinguishable from that observed for targeting saccades.

Our findings are in line with previous work that manipulated the degree of automaticity of saccade behavior by varying the predictability of the saccade target location, and observed minor or no changes of perisaccadic compression (Maij et al., 2011). Our results also agree with work comparing perisaccadic mislocalization during saccades with different levels of volitional control: “pro-saccades” targeting a peripheral onset and “anti-saccades” made in the direction opposite to the peripheral onset (Awater and Lappe, 2004). Although many saccade parameters distinguished the two types of eye movements, the patterns of perisaccadic mislocalization were markedly similar. Ostendorf et al. (2007) specifically investigated the relationship between the main saccade parameters and perisaccadic compression; while we both find that perisaccadic compression is unrelated to the distribution of saccade latency values, Ostendorf et al. (2007) found a significant correlation between perisaccadic compression and peak saccadic velocity that was not present in our dataset—nor in Maij et al. (2011).

Our second experiment may also be considered in the light of a recent study (Zimmermann et al., 2014b) that manipulated the features of the saccade target and observed the near elimination of perisaccadic compression when all visual transients associated with the target appearance were eliminated—by removing the saccade target stimulus altogether and instructing subjects to saccade to an unmarked location. One of the hypotheses put forward to explain this finding is that compression is affected by the shift of attention triggered by the sudden appearance of the saccade target. However, Zimmermann et al.’s (2014b) manipulation affected an additional factor that is known to affect localization: spatial references (Lappe et al., 2000), clearly reduced when the saccade target presentation is withheld. In this sense, our second experiment may be seen as a logical counterpart of Zimmermann et al.’s (2014b): we removed visual transients associated with the saccade target, while maintaining its role as a strong and stable visual reference. We did not find a dramatic change of compression as in Zimmermann et al. (2014b). This supports an alternative hypothesis proposed by these authors, that compression is influenced by the presence of the saccade target rather than its salient sudden appearance and the shift of attention that the latter would trigger. This is consistent with much evidence that visual factors, and specifically spatial references, play a major role in space perception at the time of eye movements (e.g., Deubel, 2004) and specifically in shaping perisaccadic mislocalization effects—as shown in a variety of paradigms that analyzed real saccades (Cicchini et al., 2013; Zimmermann et al., 2014b), simulated saccades (Ostendorf et al., 2006; Zimmermann et al., 2014a) and interrupted saccades (Atsma et al., 2014).

Like for the compression index, we find that the shift index—the other index describing perisaccadic mislocalization—is fairly well matched across conditions and experiments. However, small significant differences did emerge, which could indicate that the two indices changed independently in the contexts we examined. Two observations call for caution in interpreting these. First, note that the majority of our data is concentrated in the interval immediately following saccade onset; while at the trough of the compression index, this interval is not optimal for testing differences in the shift index, which typically peaks before the saccade onset (Lappe et al., 2000). Also note that the shift index varied markedly across observers (both for novices and experts), yet this idiosyncrasy appeared to be a consistent trait of the individual subjects, which did not normalize with practice and did not correlate with any of the saccade parameters.

These results are reassuring on the face validity of the saccadic compression phenomenon. To our knowledge, the present study is the first to follow perisaccadic mislocalization from the very first exposure until subjects master the task; finding virtually no change indicates that the phenomenon does not emerge from strategies that subjects develop *ad hoc* in this laboratory setting. This is important for two reasons. First, the experimental conditions in which compression is measured are highly unnatural—we normally don’t make saccades of constant direction and amplitude repeatedly over several minutes, and the neural substrates of saccade programming depend on the degree of automaticity of the saccade task: recruiting different areas (Johnston and Everling, 2008; McDowell et al., 2008) with a different temporal profile (Basso and Wurtz, 1997, 1998). Second, given hundreds of repetitions, subjects may develop strategies and adjust responses to cope with an unnatural context—such as the unnatural flashing of stimuli in and out of view within few milliseconds. An example of this comes from the saccade adaptation literature. Saccade adaptation is the change of saccade amplitude obtained by repeatedly displacing the target of the saccade while the latter is in-flight. Given hundreds of repetitions, this manipulation affects not only oculomotor behavior (shortening/lengthening of the saccades) but also leads to a global distortion of visual space (Zimmermann and Lappe, 2009, 2010, 2011; Schnier and Lappe, 2012)—as though the visual system had adjusted to the artificial mismatch between pre- and post-saccadic target location, incorporating it within a new and distorted spatial metrics. Our observations indicate that, on the contrary, the compressed spatial metrics representing perisaccadic flashed stimuli does not emerge as an adjustment to specific stimulus and task conditions—being present in observers that are completely new to such conditions.

In conclusion, we show that the spatial compression observed for perisaccadic flashed stimuli is a robust phenomenon, insensitive to the specific paradigm used to drive saccades and to the level of practice with the saccade task.

## Conflict of Interest Statement

The authors declare that the research was conducted in the absence of any commercial or financial relationships that could be construed as a potential conflict of interest.
